# Selective activation of primary afferent fibers evaluated by sine-wave electrical stimulation

**DOI:** 10.1186/1744-8069-1-13

**Published:** 2005-03-25

**Authors:** Kohei Koga, Hidemasa Furue, Md Harunor Rashid, Atsushi Takaki, Toshihiko Katafuchi, Megumu Yoshimura

**Affiliations:** 1 Department of Integrative Physiology, Graduate School of Medical Sciences, Kyushu University, Fukuoka 812-8582, Japan

## Abstract

Transcutaneous sine-wave stimuli at frequencies of 2000, 250 and 5 Hz (Neurometer) are thought to selectively activate Aβ, Aδ and C afferent fibers, respectively. However, there are few reports to test the selectivity of these stimuli at the cellular level. In the present study, we analyzed action potentials (APs) generated by sine-wave stimuli applied to the dorsal root in acutely isolated rat dorsal root ganglion (DRG) preparations using intracellular recordings. We also measured excitatory synaptic responses evoked by transcutaneous stimuli in substantia gelatinosa (SG) neurons of the spinal dorsal horn, which receive inputs predominantly from C and Aδ fibers, using *in vivo *patch-clamp recordings. In behavioral studies, escape or vocalization behavior of rats was observed with both 250 and 5 Hz stimuli at intensity of ~0.8 mA (T5/ T250), whereas with 2000 Hz stimulation, much higher intensity (2.14 mA, T2000) was required. In DRG neurons, APs were generated at T5/T250 by 2000 Hz stimulation in Aβ, by 250 Hz stimulation both in Aβ and Aδ, and by 5 Hz stimulation in all three classes of DRG neurons. However, the AP frequencies elicited in Aβ and Aδ by 5 Hz stimulation were much less than those reported previously in physiological condition. With *in vivo *experiments large amplitude of EPSCs in SG neurons were elicited by 250 and 5 Hz stimuli at T5/ T250. These results suggest that 2000 Hz stimulation excites selectively Aβ fibers and 5 Hz stimulation activates noxious transmission mediated mainly through C fibers. Although 250 Hz stimulation activates both Aδ and Aβ fibers, tactile sensation would not be perceived when painful sensation is produced at the same time. Therefore, 250 Hz was effective stimulus frequency for activation of Aδ fibers initiating noxious sensation. Thus, the transcutaneous sine-wave stimulation can be applied to evaluate functional changes of sensory transmission by comparing thresholds with the three stimulus frequencies.

## Background

Psychophysical procedures such as the visual analogue scale are commonly used to assess human pain magnitude. However, any of those assessments seem not to be suitable for evaluation of a change in thresholds of each class of afferent fibers, despite of the fact that quantitative analysis of each afferent class is essential for understanding underlying mechanisms of pathological states and evaluation of analgesic chemicals. It now becomes evident that hyperalgesia or allodynia produced by various chronic pain conditions is due to not only a change in threshold of fine afferent fibers but also participation of large myelinated afferents in producing allodynia in inflammation and sciatic nerve transection models [1-3]. Recently, one method that allows us to clarify a change in threshold of each afferent fiber quantitatively has been developed (Neurometer) [4]. That is a method to measure selectively the thresholds of three classes of afferent fibers by applying transcutanous sine-wave stimulation at three frequencies of 2000, 250 and 5 Hz via surface electrodes at a current intensity in the range of 0.01–9.9 mA to the skin. Neurometer is now widely used clinically to evaluate peripheral nerve sensitization and dysfunction in various painful states, including neuropathic pain or an effect of treatment of analgesic drugs [5-9]. Although several lines of evidence suggest the selective stimulation of three distinct afferent fibers by Neurometer, the validity of the method at single cellular level has not been made.

There are three types of sensory afferent fibers that send sensory information to the CNS; unmyelinated C fibers send a long lasting delayed painful sensation, thinly myelinated Aδ fibers send a short and fast painful sensation and the thickly myelinated Aβ fibers send tactile information. Several attempts have been made to identify the types of peripheral sensory fibers activated by the sine-wave stimuli using behavioral pharmacological approaches such as blockade of nerve conduction by local anesthetics or use of topical application of capsaicin, etc. [10-12]. However, no studies have been made to clarify the single neuronal properties such as action potential generation or its frequency of peripheral fibers. Moreover, responses of spinal dorsal horn neurons have not been examined by peripheral stimulation with Neurometer.

The purpose of this study was to clarify the selectivity of the sine-wave stimuli on the activation of sensory afferent fibers in detail. First, we performed behavioral tests with Neurometer to measure the withdrawal thresholds by transcutaneous application of three frequencies of stimuli. Next, we performed intracellular recordings from acutely isolated DRG neurons with attached dorsal roots to examine firing properties of C, Aδ and Aβ fibers. Moreover, using *in vivo *patch-clamp recordings, we analyzed excitatory synaptic responses evoked by the transcutaneous stimuli in SG neurons.

## Results

### Behavioral response

We first examined the escape and/or vocalization behavior of rats to transcutaneous stimuli using Neurometer. When 250 and 5 Hz stimuli were applied to the left hind limb, rats escaped from the stimuli or vocalized. The thresholds for 250 and 5 Hz stimuli were quite similar, and were averaged to be 0.74 ± 0.06 mA (T250, n = 12) and 0.84 ± 0.12 mA (T5, n = 12), respectively. Next, 2000 Hz stimulation which is used to evaluate Aβ fibers was applied. Although Aβ fibers were thought to convey innocuous information, escape and/or vocalization behavior was also observed at higher intensity. The threshold for 2000 Hz stimulation (2.14 ± 0.28 mA, T2000, n = 12) was more than two times higher than T250 and T5.

### Classification of DRG neurons

Intracellular recordings were made from 53 DRG neurons. Dorsal root stimulation with a suction electrode elicited antidromic APs with latencies in the range of 0.2 – 15.0 ms. Fast-conducting neurons had a brief action potential duration (APD), whereas slow-conducting ones had a relatively broad APD. Based on the threshold stimulus intensity (TSI), conduction velocity (CV) and APD, DRG neurons were divided into three subgroups, C, Aδ and Aβ fibers (Table 1). These results were consistent with those reported previously [1,13-17].

**Table 1:** Comparison of electrophysiological properties of DRG neurons

	RMP (mV)	CV (m/s)	TSI (mA)	APD (ms)
C fibers	-63.0 ± 1.5	0.6 ± 0.1	3.1 ± 0.5	1.38 ± 0.01
				
(n = 15)	(51.0–74.0)	(0.4–0.7)	(2.5–4.6)	(0.81–2.85)
				
				
Aδ fibers	-67.7 ± 2.4	5.9 ± 0.8	1.8 ± 0.6	0.63 ± 0.05
				
(n = 18)	(55.0–71.0)	(1.4–12.5)	(1.6–2.1)	(0.30–0.99)
				
				
Aβ fibers	-65.1 ± 1.8	19.6 ± 1.3	0.8 ± 0.2	0.29 ± 0.01
				
(n = 20)	(52.0–76.0)	(14.1–23.4)	(0.04–1.34)	(0.19–0.43)

RMP, resting membrane potential; CV, conduction velocity; TSI, threshold of stimulus intensity. APD, duration of action potential measured at its half-maximal amplitude; Values are shown as means ± SEM.

### Action potential generated in DRG neurons by sine-wave stimuli

After classification of DRG neurons, C, Aδ and Aβ neurons were stimulated by sine-wave stimuli with gradual increase in intensity using Neurometer.

#### 2000 Hz Stimulation

**Figure 1:**
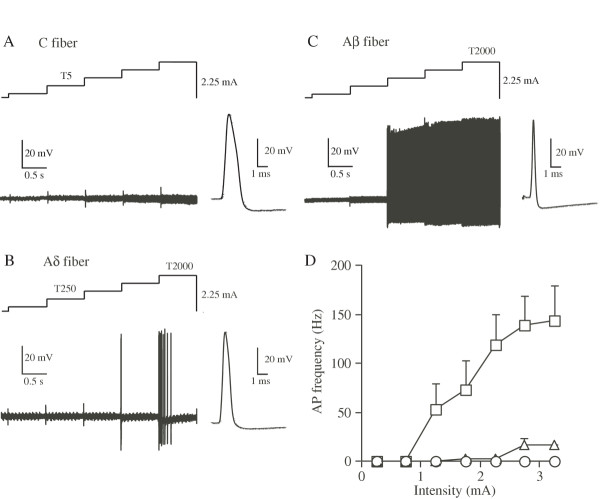
**Representative firing patterns of C (A), Aδ (B) and Aβ (C) neurons in response to 2000 Hz sine-wave stimulation. **In this and subsequent figures 2 and 3, the stimulus intensities were shown above the traces. Action potentials recorded from three types of afferent fibers were shown on the right of the traces. The frequencies of APs were plotted against the stimulus intensity (D). In this and subsequent figures of 2 and 3, ○: C fiber (n = 15), △: Aδ fiber (n = 18), □: Aβ fiber (n = 20).

As shown in figure 1A, no AP was generated even when the intensity was increased to more than 2 mA in 10 out of 15 C neurons. In the remaining 5 C neurons, one or two APs were observed at the point of intensity change at T5/T250. However, further increase in stimulus intensity did not produce any firing in these C neurons (data not shown). In all Aδ neurons tested, phasic firing with several APs was elicited at high intensities of ~2 mA (Figure 1B). On the other hand, tonic firing which lasted throughout the stimulation was produced in all Aβ neurons examined at near T5/T250 (Figure 1C). Figure 1D demonstrates the AP frequency plotted against stimulus intensity. The AP frequency of C neurons was near zero at intensities less than 3 mA. In Aδ neurons, AP was elicited at 1.3 mA and the frequency was increased to ~17 Hz at 2.8 mA. In Aβ neurons, AP was generated at 0.8 mA and the frequency increased steeply and reached ~140 Hz at the intensity of 2.7 mA. These data indicate that 2000 Hz stimulation at low intensity (near T5/250) selectively activates Aβ neurons.

#### 250 Hz Stimulation

**Figure 2:**
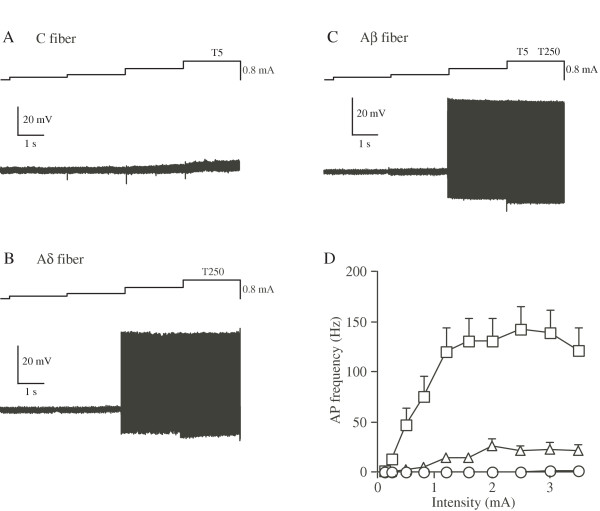
**Representative firing patterns of C (A), Aδ (B) and Aβ (C) neurons in response to 250 Hz sine-wave stimulation. **The frequencies of APs were plotted against the stimulus intensity (D).

250 Hz stimulation at intensity below T5/T250 initiated APs in Aδ and Aβ neurons but not in C neurons examined (Figures 2A–C). As shown in figures 2D, the AP frequency of Aβ and Aδ neurons started to increase from 0.3 mA and reached plateau at 2 mA.

#### 5 Hz Stimulation

**Figure 3:**
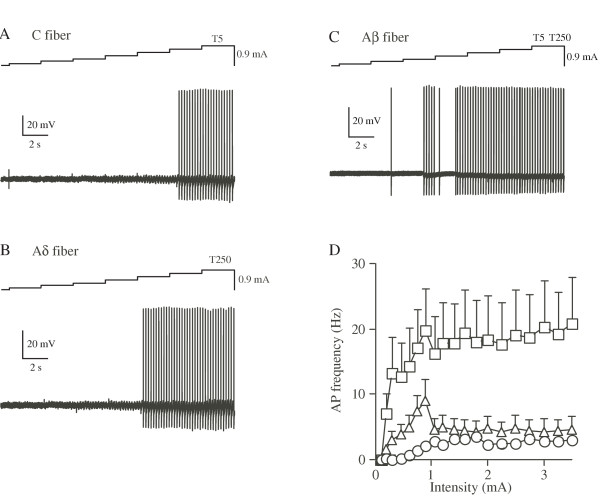
**Representative firing patterns of C (A), Aδ (B) and Aβ (C) neurons in response to 5 Hz sine-wave stimulation. **The frequencies of APs were plotted against the stimulus intensity (D).

5 Hz stimulation produced tonic firing in all three classes of DRG neurons examined (Figures 3A–C). As shown figure 3D, in C neurons, APs were elicited at 0.4 mA and the frequency reached plateau at ~1 mA. In Aδ neurons, APs were generated at 0.3 mA and the frequency reached the maximum at 0.8 mA. In Aβ neurons, APs were produced at 0.1 mA and the frequency reached plateau at 0.2 mA. Taken together these three studies shown in figures 1,2,3, the data indicate that C fibers are activated only by 5 Hz stimulation among the three specific frequencies.

### Excitatory synaptic responses evoked in SG neurons *in vivo *by cutaneous noxious stimuli

**Figure 4:**
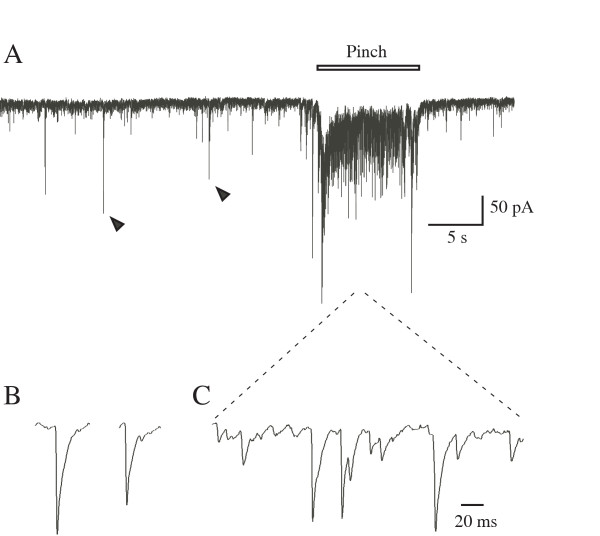
**EPSCs in SG neurons occurring spontaneously and in response to pinch stimulation *in vivo *(A). **Pinch stimulation was applied to the skin of the hind limb. EPSCs indicated by arrowheads and pinch-evoked EPSCs were shown an expanded time scale in B and C, respectively.

It is well known that SG neurons in the spinal dorsal horn receive synaptic inputs predominantly from C and Aδ fibers [18-20]. Therefore, SG is thought to be specifically implicated in nociceptive processing. As shown previously [21-24], SG neurons exhibited miniature and spontaneous excitatory postsynaptic currents (mEPSCs and sEPSC, respectively) under voltage-clamp condition at a holding potential of -70 mV. The large amplitude of EPSCs were reversibly blocked by TTX (1 μM) applied to the surface of the spinal cord leaving mEPSCs intact (data not shown). Therefore, the large amplitude of EPSCs (sEPSCs) were mediated by spontaneous firings of primary afferents or interneurons. Pinch stimulation applied to the skin of the hind limb produced a barrage of EPSCs (defined as evoked EPSCs; eEPSCs) in all 24 SG neurons examined (Figure 4).

### Excitatory synaptic responses evoked in SG neurons *in vivo *by transcutaneous sine-wave stimuli

**Figure 5:**
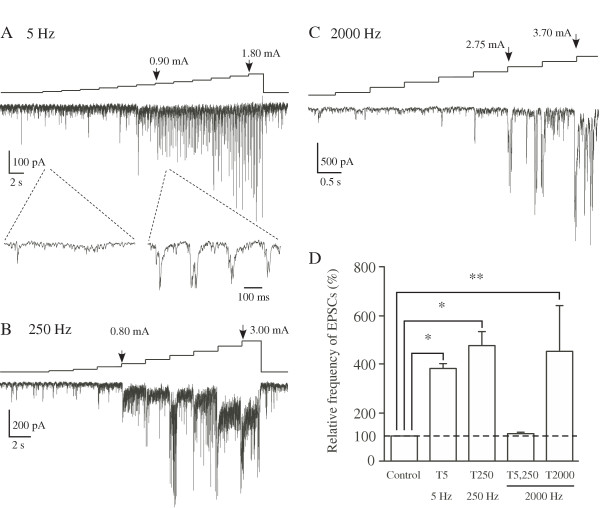
**EPSCs in SG neurons evoked by 5 (A), 250 (B) and 2000 (C) Hz transcutaneous sine-wave stimuli. **In A-C, the stimulus intensities were shown above the traces. Lower two records in *A *were shown in an expanded time scale. Relative frequencies of large amplitude of EPSCs evoked by the stimuli at intensities of T5, T250 or T 2000 (D). *P < 0.05, **P < 0.01.

**Figure 6:**
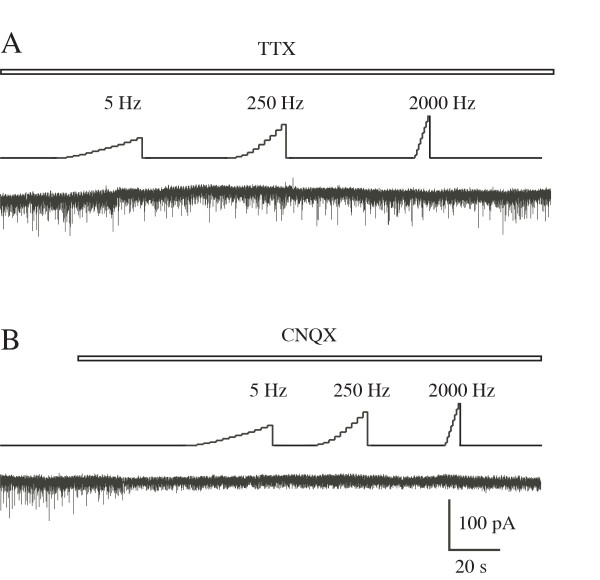
EPSCs in SG neurons recorded in the presence of TTX (1 μM, A) or CNQX (20 μM, B).

When the sine-wave stimuli was applied to the skin of the receptive field, 250 and 5 Hz stimuli evoked a barrage of EPSCs at a stimulus intensity of ~0.8 mA (Figures 5A and 5B). On the other hand, SG neurons showed an increase in frequency of large amplitude of EPSCs with 2000 Hz stimulation at higher intensities (Figure 5C) in spite of few inputs from Aβ fibers to SG, suggesting that the EPSCs elicited by 2000 Hz stimulation were mediated by the activation of Aδ afferents. Figure 5D shows the frequency of eEPSCs (> 50 pA) evoked at the behavioral thresholds of the three stimuli. The average frequency of 5 Hz-evoked EPSCs at the behavioral threshold was increased to 378 ± 30 % of control (1.8 ± 0.3 Hz, n = 7) and that of 250 Hz-evoked EPSCs to 473 ± 69 % (2.9 ± 0.7 Hz, n = 8), and that of 2000 Hz-evoked EPSCs to 602 ± 198 % (2.9 ± 0.8 Hz at the behavioral threshold T2000, n = 6). The large amplitude EPSCs evoked by sine-wave stimuli were blocked by TTX (1 μM) and all EPSCs were abolished by a glutamatergic AMPA receptor antagonist CNQX (20 μM; Figures 6A and 6B).

## Discussion

The present study demonstrates that 2000 Hz sine-wave stimulation activates selectively Aβ fibers, and 250 and 5 Hz stimuli excite noxious transmission to SG neurons mediated mainly through Aδ and C fibers, respectively, though 250 Hz stimulation also activates Aβ fibers. Our study is the first to examine the selective activation of sensory transmission by the sine-wave stimuli at the cellular level, and the results support that transcutaneous sine-wave stimuli can be applied to examine clinically the changes of threshold in each subtype of afferent fibers following pathological conditions or after treatment with analgesics based on the combination of three sine-wave stimuli.

### Activation of nociceptive transmission by sine-wave stimulation

We observed that the stimulus thresholds producing the escape and/or vocalization behavior in rats with 250 and 5 Hz stimuli were not significantly different, whereas the threshold for 2000 Hz stimulation was two times higher than those of 250 and 5 Hz stimuli. The values of these thresholds are consistent with those of previous study [10]. These observations suggest that transcutaneous stimuli at frequency of 250 and 5 Hz induce pain sensation, and the 2000 Hz stimulation may also induce pain at higher intensity. From the analysis in C and Aδ fibers, which predominantly convey noxious information, we found that 5 Hz stimuli activated both C and Aδ fibers, 250 Hz stimulation activated only Aδ at low intensity (~0.8 mA). Interestingly, Aδ fibers were also activated by 2000 Hz stimulation, although 2000 Hz stimulation is thought to activate Aβ fibers. However, the activation of Aδ fibers by 2000 Hz was observed only at high stimulus intensity (> 2 mA). In *in vivo *analysis of EPSCs evoked in SG neurons, which receive monosynaptic inputs predominantly from C and Aδ fibers [18-20], we found that 2000, 250 and 5 Hz stimuli significantly increased the frequency of evoked EPSCs at their behavioral thresholds. Therefore, these results indicate that 250 and 5 Hz stimuli, produce nociceptive responses by activation of Aδ fibers and by activation of C and Aδ fibers, respectively. On the other hand, 2000 Hz stimulation at high intensity could activate Aδ fibers and thus initiate noxious sensation. There was no significant difference in the frequencies of EPSCs evoked by 250, 5 and 2000 Hz stimuli at the behavioral threshold (Figure 5D). In the previous behavioral pharmacological study, topical application of capsaicin to the skin, a specific desensitizer of small fibers [25], increased behavioral thresholds for 250 and 5 Hz but not 2000 Hz stimuli, indicating that 250 and 5 Hz stimuli activate small fibers [10]. This observation is consistent with our results.

### Discharge ability of sensory fibers

We observed that C fibers were activated only by 5 Hz stimulation with a maximum frequency of ~3 Hz, Aδ fibers were activated by 250 and 5 Hz (maximum frequency of ~22 Hz with 250 Hz stimulation) and Aβ fibers were activated by all three stimuli (maximum frequency of ~140 Hz with 2000 Hz stimulation, Figures 1,2,3). One of the most important factors for affecting the maximum frequency of sensory fibers is the inactivation of voltage-dependent sodium channels. In other words, discharge frequency of fibers is controlled by the time taken to recover the sodium channels from their inactivation state. Recent studies have shown that TTX-sensitive sodium channels expressed in large diameter DRG neurons exhibit faster recovery from the inactivation than the TTX-resistant ones expressed in small DRG neurons [26-29]. Therefore, it can be speculated that high frequency sine-wave stimuli are unable to initiate APs in small C fibers due to slow repriming of TTX-resistant sodium channels.

Passive membrane property is also an important factor for the ability of neurons to discharge. Previous report has shown that the membrane properties of the three subgroups of rat DRG neurons were varied [17]. In particular, the membrane time constant of C neurons (6.5 ms) is much longer than those of Aδ (2.6 ms) and Aβ (1.8 ms) neurons. On the other hand, the durations of single sine-wave stimuli at frequencies of 2000 Hz and 250 Hz are 0.5 ms and 4 ms, respectively. The membrane time constant is the time required for the voltage change across the membrane to reach ~63% of its final value [30]. Therefore, the longer membrane time constant might contribute to the failure of AP in C neurons with 2000 and 250 Hz stimuli, since the membrane potential is slowly depolarized, resulting that the membrane potential can not reach AP threshold within the short depolarizing cycles with the high frequency stimuli as compared to 5 Hz one. In C fiber of rat DRG neurons, AP failure was increased when repetitive stimulation was applied at high frequencies [1]. Consistent with the lines of evidence, in the present study, there was no firing in most C fibers with 250 Hz and 2000 Hz stimuli even when the high intensity stimuli were applied as described in the results.

### Selectivity of specific frequencies of sine-wave stimuli to activate primary afferents

#### 2000 Hz Stimulation

We found that 2000 Hz stimulation selectively activated Aβ fibers at low intensity (< 2 mA). Only at higher intensities (> 2 mA), 2000 Hz stimulation produced APs in Aδ fibers. On the other hand, 2000 Hz stimulation increased EPSC frequency in SG neurons, which are known to receive a few Aβ inputs, at higher stimulus intensity. Therefore, it is conceivable that EPSCs in SG neurons by 2000 Hz stimulation at higher intensities are produced predominantly by activation of Aδ fibers.

#### 250 Hz Stimulation

250 Hz stimulation produced APs in Aδ fibers (with a max discharge frequency of 22.3 ± 5.9 Hz) and Aβ fibers (with a max discharge frequency of 133.4 ± 13.3 Hz). These values of AP frequencies were consistent with those evoked by physiological stimuli [31]. These results suggest that 250 Hz stimulation initiates both nociceptive sensation via Aδ fibers and tactile sensation via Aβ fibers. However, in general when both painful and tactile sensations are produced simultaneously, the painful sensation would only be perceived. Therefore, 250 Hz is thought to be effective stimulus frequency for activation of Aδ fibers initiating noxious sensation.

#### 5 Hz Stimulation

The present study showed that C fibers were activated only by 5 Hz stimulation, but not by other stimulus frequencies. This indicates that 5 Hz stimulation is effective to activate C fibers among the three specific frequencies. Although both Aδ and Aβ fibers were also activated by 5 Hz stimulation, it is conceivable that the activation of Aδ or Aβ fibers by 5 Hz stimulation does not reach sufficient frequency to induce functional sensation. In the present study with 5 Hz stimulation, the maximum AP frequencies were ~3 Hz in C fibers, ~4 Hz in Aδ fibers and ~20 Hz in Aβ fibers (Figures 1,2,3). On the other hand, it has been reported that AP frequencies produced by natural physiological stimuli in rat DRG neurons are: ~5 Hz in C fibers by mechanical nociceptive stimuli, ~15 Hz in Aδ fibers by high threshold mechanical stimuli and ~100 Hz in Aβ fibers by slowly adapting tactile stimuli [31]. Although the AP frequency by 5 Hz stimulation in C fibers in our results was almost similar to the value of AP frequency in C fibers by natural mechanical noxious stimulation, the AP frequencies in Aδ and Aβ fibers by the 5 Hz stimulation were much less than those produced by natural stimuli. Furthermore, the number of C fibers in rat DRG neurons is quite larger than those of Aδ and Aβ fibers (C fiber, ~63%; Aδ fiber; ~25%; Aβ fiber, ~12%) [32].

**Figure 7:**
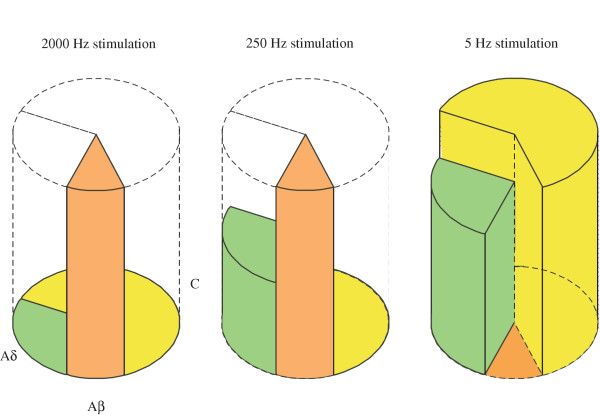
**Schematic diagram of activation of afferent fibers with sine-wave stimuli. **The pie showed the number of three types of rat DRG neurons as reported by Djouhri et al [32]. The height represented normalized AP frequencies produced by sine-wave stimuli in the three types of fibers in our study relative to those by natural stimuli reported previously [31]. This figure shows the selectivity of the sine-wave stimuli to activate afferent fibers as described in 'Discussion'.

Based on the present observations, the following analysis could be made (Figure 7). 2000 Hz sine-wave stimulation selectively activates Aβ afferents. This result could make it possible to analyze a change in threshold of Aβ afferent. If this stimulation protocol could produce pain sensation, it would be suggested that tactile stimuli produce pain, i.e. allodynia. 250 Hz stimulation activates both Aδ and Aβ fibers. In combination with 2000 Hz stimulation, a change of threshold in Aδ afferent could be examined. However, a substantial number of Aδ afferents convey non-noxious sensation, and therefore, it will be difficult to differentiate convincingly whether a change in sensitivity of Aδ afferent causes allodynia or hyperalgesia. Although, 5 Hz stimulation activates not only C but also Aδ and Aβ afferents, a change in C afferent threshold could be analyzed by excluding Aβ and Aδ afferent changes which can be performed by both 2000 and 250 Hz stimuli.

In conclusion, we provided electrophysiological evidence that the sine-wave stimulation could differentiate changes in threshold of Aβ, Aδ and C afferent fibers by combining data obtained from 2000, 250 and 5 Hz stimulus protocol. This analysis using Neurometer would be useful for understanding not only changes in primary afferent fibers due to pathological conditions but also the evaluation of clinical treatment of analgesics.

## Methods

All experiments involving the *in vivo *study were approved by the Committee of the Ethics on Animal Experiments of Kyushu University and conducted in accordance with the Guiding Principles of the Care and Use of Animals in the Field of Physiological Science of the Physiological Society of Japan.

### Animals and Behavioral test

Male Sprague-Dawley rats (6–9-week old), weighing 250–300 g were used for behavioral tests, intracellular recordings and *in vivo *patch-clamp recordings. A skin patch dispersion electrode was fixed to attach the skin of left hind limb of rats. Transcutaneous nerve stimuli were applied through this electrode using the nociceptive mode of the Neurometer^® ^(NUROTRON INCORPORATED, Baltimore, USA). In this mode, the intensity was automatically increased in step from 0 to 9.99 mA (29 steps for 5 Hz stimulation, 20 steps for 250 Hz and 2000 Hz stimuli). The durations were 2.5 sec for 5 Hz and 2.1 sec for 250 Hz and 0.72 sec for 2000 Hz stimuli. When rats escaped and/or vocalized, the stimulation was immediately stopped and the stimulus intensity was defined as behavioral threshold.

### Intracellular recording from DRG neurons

The methods used for the current experiment were similar to those described previously [1]. Under anesthesia with diethyl ether, a rat was decapitated and the lumbar laminectomy was performed. L4-6 DRGs with an attached proximal dorsal root were isolated from the animal. The isolated DRG was submerged in Krebs solution (in mM: NaCl 117, KCl 3.6, CaCl_2 _2.5, MgCl_2 _1.2, NaH_2_PO_4 _1.2, NaHCO_3 _2.5 and glucose 11) equilibrated with 95% O_2_-5% CO_2 _and maintained at 36 ± 1°C and set in a recording chamber. Intracellular recordings of APs were made from DRG neurons with glass-microelectrodes having a DC tip resistance of 50–100 MΩ, filled with 4 M potassium-acetate. Signals were amplified with a high input-impedance bridge amplifier (Axoclamp 2B; Axon Instruments, Foster City CA, USA). Artifacts were minimized with low-pass (1000 Hz) or notch (250 Hz) filters using pCLAMP program (Axon Instruments). Data from neurons with resting membrane potentials less than – 50 mV and AP amplitudes smaller than 60 mV were excluded in the present study. AP duration was determined at half of the peak amplitude of the AP. Antidromic stimulation (duration 100 μsec) was given to the central end of the dorsal root with a suction electrode. The stimulus intensity was monitored with a digitized output isolator (ss-202J; Nihon Kohden, Tokyo, Japan). The value of conduction velocity was calculated from the latency of AP and the length of the dorsal root retained.

### *In vivo *patch-clamp recording from SG in dorsal horn

The methods for *in vivo *patch-clamp recording from SG neurons have been described in detailed elsewhere [22,24]. Briefly, under artificial ventilation, a lumbar laminectomy was performed at the level of L4–L5 and the animal was then placed in a stereotaxic apparatus (Model ST-7, Narishige, Japan). Under a binocular microscope with ×8 ~ × 40 magnification, the dura mater was cut and reflected, and then either L4 or L5 dorsal root was shifted laterally using a glass hook. The pia-arachnoid membrane was cut to make a window to allow the patch electrode into the spinal cord. The patch pipette had a tip resistance of 8–10 MΩ when filled with a solution of the following composition (in mM): Cs_2_SO_4 _110, CaCl_2 _0.5, MgCl_2 _2, EGTA 5, HEPES 5, Mg-ATP 5, tetraethylammonium 5. Signals were acquired with an Axopatch 200B amplifier (Axon Instruments), low-pass-filtered at 5 kHz. Data were analyzed using Axograph program (Axon Instruments). The surface of the exposed spinal cord was perfused with Krebs solution equilibrated with 95% O_2_-5% CO_2 _at 38 ± 0.5°C. Drugs were dissolved in Krebs solution and applied to the perfusing line. Whole-cell patch-clamp recordings could be obtained from *in vivo *preparations for more than 8 hours and stable recordings were made from single SG neurons for up to 2 hours. After the end of the experiments the animals were killed by exanguination.

### Statistical analysis

Data were presented as mean ± SEM. Statistical significance was determined as *P *< 0.05 using Dunnett's multiple comparison.
